# Tuning into Harmut Rosa’s systematic romanticism

**DOI:** 10.1186/s40711-023-00189-2

**Published:** 2023-06-02

**Authors:** Frédéric Vandenberghe

**Affiliations:** 1 Federal University of Rio de Janeiro, Rio de Janeiro, Brazil; 2 Max Weber Kolleg, University of Erfurt, Erfurt, Germany

**Keywords:** Hartmut Rosa, Charles Taylor, Acceleration, Alienation, Resonance

## Abstract

The article reconstructs the intellectual itinerary of the German social theorist Hartmut Rosa. It follows the development of his oeuvre, from his doctoral thesis on Charles Taylor and his book on social acceleration to his more recent work on resonance and responsivity. It shows that throughout the four phases of his career, the social philosophy of Charles Taylor has had a decisive influence on his philosophical anthropology, theory of society and moral sociology. It calls for a new rapprochement between the different generations of critical theory to think through societal pathologies without giving up on the promises of modernity.

## Introduction: reconstruction and critique

With four successive generations of scholars, the Frankfurt School is now almost a centennial school of thought. From within the fold of critical theory a new star has risen at the intellectual firmament: Hartmut Rosa.^1^ His work is widely discussed in Germany, the Anglosphere, Latin America and now also in China. Rosa has a knack for choosing large transversal topics that allow him to interweave broad theoretical discussions in philosophy and sociology with more existential issues. If his readers are interested and moved by his work, it is because he’s able to connect the topics he puts on the agenda for reflection – moral maps, acceleration, alienation, and resonance—to their personal life. A glance at the secondary literature on his work shows that his name is almost automatically associated with “acceleration” and “resonance”. *Social Acceleration* (2005) and *Resonance* (2016), his major theoretical works so far, open up large vistas on conceptual landscapes in critical social theory that blend a radical critique of large-scale social systems that are out of control with a more romantic yearning for social integration, cultural significance and personal connection. It is not easy to combine a systemic critique of modernity with more existential issues, but that is what defines Rosa’s work.

In this article, I want to present a systematic and critical reconstruction of Hartmut Rosa’s work.^2^ While the reconstructive part suggests one should look at the moral and political philosophy of Charles Taylor to understand Rosa’s social theory, the critical part intimates that the weaknesses of his approach stem from this same filiation. For starters, I will argue that to fully understand and appreciate his work, one should take into account his first (untranslated) book *Identity and Cultural Praxis* (1998). This book originates in a Ph.D. on the social philosophy of Charles Taylor he defended at the Humboldt University in Berlin in 1996 under the guidance of Axel Honneth. He asks the question “What holds the work of Charles Taylor as a whole together?” (Rosa 1998: 72), and answers it with a reference to the philosophical anthropology that traverses and structures his critique of behaviourism, his hermeneutical philosophy of language, his existential phenomenology, his theory of human agency, his cultural genealogy of the modern self, his politics of recognition, his communitarian critique of liberalism and his theological reflections on secularism. In Taylor, philosophical anthropology comes in two complementary versions: a more fundamental one that spells out in quasi-transcendental fashion what it means to be a human being (analysed in the first part of the Ph.D.) and a second, more historical one, that explores what it means to be a modern human being (analysed in the second part of the Ph.D.). Rosa’s dialogical reconstruction of Taylor’s trajectory is systematic. It shows that the formation of a stable personal identity presupposes a cultural background of shared worldviews and values—hence the title. It is also critical. It points to an unresolved rift in Taylor’s work between his realism and constructivism, universalism and relativism, essentialism and historicism, and some solutions (in the third and last part of the Ph.D.).

My own reconstruction of Rosa’s work will follow his trail and alternate between exposition and critique. In echo to his Ph.D., I will ask, “What holds the work of Hartmut Rosa as a whole together?” My answer will be that throughout his career the reference to the inspirational work of Charles Taylor is constant and that it forms the undertow of his whole oeuvre. Taylor is for Rosa what Hegel is for Taylor. A good deal of his intellectual motives and themes, including resonance (Goldstein 2018), comes directly from Taylor. I will follow a chronological line, distinguish four phases in his career and show the influence of Taylor in each and throughout all the phases. In a first, formative phase (1994–2001), Rosa will engage Taylor´s philosophical anthropology, intellectual history, moral philosophy and communitarian politics and lay the foundations of a critical hermeneutics of the self and a communitarian critique of modernity. In the second phase of his work (2001–2009) on the temporal structures, processes and practices of early, classic and late modernity, he will transform the metaphor of acceleration into a wide-ranging, all-encompassing prophetic analysis and critical diagnosis of the times, according to which the dialectic of the Enlightenment has come to a “frenetic standstill”. His chronosophical diagnosis of post-modern, post-industrial and neo-liberal societies systematically thinks through and works out Taylor’s moral critique of the “growth machine” in an epochalist theory of high-speed societies. In the third phase (2009–2011), hitting middle age, the German theorist will return to his early interest in moral philosophy and complement his sociological diagnosis of acceleration with a normative critique of alienation. Like the predecessors of the first generation of the Frankfurt School, he will radicalise his critique of industrial capitalism, denounce alienation as an anthropological catastrophe and put the “question of an alternative to modernity” (2009a: 33) on the agenda. Frightened as it were by the radicalism of his own negative conclusions, in a fourth and last phase (2011-present), Rosa will develop resonance theory as a hermeneutically sensitive, phenomenologically inspired, moral sociology of fulfilling relations to the world that complements the critical theory of alienation and reification with an affirmative philosophical anthropology. The engagement with resonance will mark a return to the philosophical anthropology and moral philosophy of the first phase. It is accompanied by a double shift of emphasis, first, from the critique of temporal structures to the critique of alienated world-relations and, secondly, from a negative “entropology” to a positive philosophical anthropology that opens up the way to an “affirmative revolution” (Rosa 2017a, b).

Throughout the four phases of his journey through social philosophy (phase 1), the theory of society (phase 2), critical theory (phase 3) and moral sociology (phase 4), Taylor’s work has been a constant source of inspiration. Once the philosophical orientation to self and society, nature and culture, modernity and post-modernity is understood, one can show how it reverberates in the four domains of interest that traverse all of his books. The positions he defends in philosophical anthropology, moral philosophy and social theory converge in a “strong social philosophy” (1998: 22, see also 548 sq.) that ties together substantive conceptions of identity, culture, society and politics in a communitarian sociology of the good life in late modernity.

In a slightly more critical vein, I will argue against this initial choice. If Rosa had chosen Paul Ricoeur, Axel Honneth or Jürgen Habermas as his anchor to explore the social, cultural and historical conditions of successful identity formation in contemporary societies, the continuity with the project of critical theory would have been easier. This is not just a question of a personal intellectual preference for the conceptual density of the continental traditions of hermeneutical phenomenology (Ricoeur) and critical theory (Habermas and Honneth) over the conceptual clarity (but at the price of vacuity?) of the analytic tradition in social, moral and political philosophy. Indeed, I think that most of the problems of Rosa’s moral philosophy, social theory and critical diagnosis of modernity stem from his alignment to Charles Taylor’s communitarian and romantic vision of society, politics, culture and identity in modern times. His systematic reconstruction of the social philosophy of Charles Taylor is sympathetic, albeit not uncritical. He clearly puts his finger on the unresolved tension between a philosophical and a historical anthropology, between a universalist conception of human capabilities and a more contextual understanding of historical identities that traverses his oeuvre. But he does not really question the parochialism of his communitarianism, the provincialism of his patriotism or the sentimentalism of his Catholicism. As his critique of Taylor is reconstructive and immanent, he does not sufficiently clear its axiological horizon from a lingering moral conservatism and anti-modernist nostalgia.

Like the first Frankfurt School, the radical critique of modernity overshoots its mark. His Rosa’s time-diagnosis of the crises of desynchronisation and the pathologies of acceleration reveals a latent anti-modernism and a patent romanticism. The opposition between autonomy and authenticity and, then, later, between alienation and resonance, is too stark. His critique of autonomy exposes not only his political “illiberalism”, but also the conceptual problems that arise when one disconnects the critique of society from the critique of practical reason, in the Kantian sense of the term.

What distinguishes a critical theory of social pathologies from hermeneutics is the belief that (in spite of everything) reason remains operative in history as a force. This belief in reason, which connects the first to the second and the third generation of the Frankfurt School, allows one to connect an immanent critique of society in its own terms to a context-transcending critique in the name of reason (Honneth 2007). By giving up on reason, re-infusing intuition and sentiment into the analysis, Rosa reverts to a romantic critique of modernity and alienation. The vagueness of his proposals for a convivialist politics for a “post-growth society” -degrowth? economic democracy? ethical consumption? basic income? complementary currencies? (Rosa and Henning 2018)—betrays that in the end his critique is not so much a social critique of capitalism as an “artistic critique” (Boltanski and Chiapello 1999) of alienation in modernity that rehabilitates the clichés of the nineteenth and twentieth century’s *Kulturkritik* of moral decadence (see also the debate in Dörre et al. 2009).

In this article, I will present Rosa´s work as an ongoing engagement with the social philosophy of Charles Taylor. The transition from moral and political philosophy to social theory and critical theory is a fluid one. While Taylor is a professional philosopher in the analytic tradition with profound knowledge of the continental tradition, Rosa is a classical social theorist who places himself in the tradition of critical theory. Both are public intellectuals on the Left with ecological sympathies, romantic leanings and religious sensibilities. Both are also interested in the cultural preconditions of the formation of individual and collective identities and are worried about the depletion of cultural resources in advanced modern societies.

In Taylor’s work, which can be considered an analytic rendering of Hegel’s social philosophy via the detour of intellectual history, one finds two interconnected, but heterogeneous and contradictory strands of social self-interpretation that are constitutive of modern identity. The two strands were already summarily mapped in his great book on Hegel (Taylor 1975: 3–50); they will be developed, refined and expanded in *Sources of the Self* (Taylor 1989) and *The Secular Age* (Taylor 2007). The first one is naturalist, instrumental and utilitarian. It values objectivity, autonomy and control. It constitutes the dominant master frame of modernity. The second one is romantic. It values subjectivity, authenticity and self-expression. While the first strand values “radical freedom” (Taylor 1975: 33), the second one treasures “integral expression” (id.). In his book on the philosophy of Charles Taylor, Rosa opposes naturalism and expressivism as two conflicting paradigms of society and identity. *Social Acceleration* shows the dead ends of a culture of autonomy and control. The necessity to synchronise practices at all levels and in all spheres of life has spawned reified systems that are out of control, undermine autonomy and alienate subjects. *Resonance* follows the second strand and opposes the expressive-mimetic relation of self to the world to the non-relation of alienation. It is only by taking the three works together that Rosa’s full intellectual landscape becomes visible. Before one can see the whole, one must, however, look at the parts and analyse them serially and sequentially.

## The philosophical and historical anthropology of Charles Taylor

### Being human 1: transcendental analytics

Rosa’s doctoral thesis is all about the relations between “moral maps”, “moral cards”  and personal identity. It contains *in nuce* most of the themes and issues of his later work. The book opens with an epigraph from “Lonesome”, a poem by the young Nietzsche on the torments of a homeless mind, uprooted, lonely and estranged in a disenchanted world, that will reappear at a later stage of his work (2017: 533). Because of reification, this world has been transformed in a “desert, mute and cold”. This world without resonance serves as the backdrop for a multifaceted exploration of the philosophical anthropology of Charles Taylor, which is centrally concerned with the moral landscapes of humanity—the “place of Man in the cosmos”, as Max Scheler (1976) phrases it in the foundational text of philosophical anthropology- and the formation of identity in modern times.

According to Rosa (1998: 72), the question: “What is Man?”—or better: “What is it like to be a human being?” is “the central and unifying theme” of Taylor´s social philosophy. For any social and political theory, this question is both unavoidable (“terribly necessary”) and utterly dubious (“unbearably problematic”, as Taylor (1988: vii) says). In the wake of post-structuralism and feminism, it is indeed no longer possible to invoke the “essence of Man” as a transhistorical substance. Although any possible answer unavoidably has to take into account the historical, social and cultural variability and situatedness of human beings, the question can, however, be meaningfully reformulated from a phenomenological-existential standpoint as a question about the general conditions that make it possible to be a human agent and a person. Taylor spells out his vision of the Anthropos by means of three interrelated concepts: “Self-interpretation”, “Strong Evaluation” and “Articulation”.

### Self-interpretation

In Taylor’s work, the answer to the question what is it means to be human takes the form of a “Best Account”. According to the “BA Principle” (Taylor 1989: 58–59), one cannot describe what a human being is from a third person perspective (like yesterday´s behaviourism or today´s neuro-cognitivism) without “changing the subject”. To understand what it means to be human, explanations must be “adequate on the level of meaning” (Weber). One must thus adopt an “emic” perspective, reconstruct the self-understandings of the actors from within and take seriously their moral intuitions. Like other authors in the phenomenological and critical tradition who have also taken the “linguistic turn”, be it with Heidegger (Gadamer and Ricoeur) or with Wittgenstein (Apel and Habermas), Charles Taylor assumes that life is always already “pre-interpreted” and “pre-understood”.

*Verstehen* is therefore not a method, but an ontological way of being in the world. In the tradition of post-Heideggerian hermeneutics, human life always and inevitably takes place in the “clearing” (*Lichtung*) that opens up and discloses the world as a human world, a world in which the environment has significance and is endowed with meaning and value. It is because as “self-interpreting animals” (Taylor 1985a: 45–76) we give value to our environment that it makes sense. The meanings that orient action vary from culture to culture, but in each case, they configure the space of possible self-interpretations in which actions occur. The interpretations of the world are of the “second order”. As the world is always already interpreted, they are interpretations of interpretations. “What is interpreted is itself an interpretation”, says Taylor (1985b: 26); “a self-interpretation which is embedded in a stream of action”. Like in Clifford Geertz’s famous Indian story about elephants that sustain turtles, interpretations go thus “all the way down”; we can now add that, in Charles Taylor and Hartmut Rosa, they also go “all the way up” as they lighten up the space of meanings and values in which human beings appear as human agents that are driven to their higher self by cultural worldviews, moral motivations and spiritual aspirations.

### Strong evaluation

One of Taylor’s central claims is that human self-understandings and modes of action are motivated by evaluative moral frameworks that define the standards by which subjects judge their life meaningful, valuable and good, and construct their identity. The values a community espouses and the ideals it cherishes configure the moral choices and the personal identities of its members. Over and over again, Rosa underscores that one’s identity is determined, in the last instance, by the fact that one is positioned in a “moral space” of common meanings and values and, in the first instance, by one’s personal “moral maps” that give meaning and direction to one’s life as a whole. To become who one truly is, one must situate oneself in the “moral space” that defines a community’s values and personalise it by crafting “moral maps” that allow one to locate oneself in relation to what is considered good or bad, worthy or unworthy, lofty or depraved.

Based on Harry Frankfurt’s concept of “second order desires”, Taylor (1985a: 15–16, 102) introduces the concept of “strong evaluations” to refer to a reflexive ordering of desires that expresses what the person really values and cares about. Let us take an example from a tourist who visits the Red light district in Amsterdam: A beautiful woman in the window hails John. Although he is tempted by his “first order desire”, he decides it would be unworthy of him, degrading to the woman in the window and his wife at home to give in to his lust. His moral aspirations to be a good husband and a decent human being define what is possible for him and orient his actions. By means of strong evaluations people define their ultimate concerns in life and, thereby, also their personal identity. Taylor can hardly imagine human beings without strong evaluations that orient their identity and, mediately, also their actions. Without the moral cartograms that define their better selves and higher angels (pinpoints on the map), individuals would simply be lost in moral space (Löw-Beer 1991)– adrift in a meaningless world, transcendentally homeless and alienated from themselves.

### Articulation

Taylor’s philosophical anthropology conjoins a cultural hermeneutics and a moral phenomenology into a transcendental inquiry into the cultural conditions of successful self-realisation. It reveals a “double hermeneutics” (Giddens 1982: 1–17) between the collective repertoires of self-description and self-evaluation on the one hand and the personal selection of moral maps that allow a subject to orient oneself in life on the other. To be human, one needs a self; as “one is a self only among other selves” (Taylor 1989: 35), one also needs others. One needs to be inserted in “webs of interlocution” and interiorise, as well as personalise, the values and ideals that the community puts at one’s disposal. The relation between the collective (the “We”) and the personal (the “I”) is not one of determination. Rather, like in the case of *langue* and *parole* (Saussure), *Sprache* and *Gespräch* (Heidegger) or form of life and practice (Wittgenstein), the relation is one of co-constitution.

Both at the individual and the collective level, the self-descriptions and self-evaluations may be inchoate and implicit or articulated and explicit. In “Four Levels of Self-Interpretation” (2012: 104–147), an important synthetic article on interpretation, articulation and critique, the young scholar elaborates a hermeneutic paradigm for social philosophy and political criticism that distinguishes four levels of self- interpretation (one implicit and the other explicit, either at the individual or at the collective level). One of the tasks of the intellectual—and remember, at the end of the day, everybody is an intellectual- is to “articulate” the tacit background of moral values and social practices by making it explicit and bringing it into language. At the individual level, emotions may dimly express values and meanings that need to be articulated to become fully explicit and conscious. At the collective level, they may exist in embodied practices (habitus) and institutions or they may be articulated in language and find their full expression in religion, philosophy, the arts and the sciences. Together, the implicit self-interpretations (institutions, habits and body-practices) and the explicit self-descriptions of society that orient conduct at both the individual and collective level form the “objective spirit” of society.

By bringing emotions, meanings and values that underlie practices into language, the array of goods to which individuals and communities adhere can be articulated, elaborated and submitted to discussion. Rosa (1994) follows the history of ideas of the Cambridge School (Pocock and Skinner, both of whom were influenced by Taylor and whose work he had discovered during his stay at the London School of Economics and covered in his Master’s thesis). With Terence Ball’s (1998) critical hermeneutics, he assumes that cultural anthropologies, social theories and political philosophies can help to articulate the self-consciousness of a society and actuate social, cultural and personal morphogenesis. In any case, the dialogical interplay between explicit (theories) and implicit self-understandings (practices) is what drives both personal and social history, according to Rosa. Crosscutting three dimensions (individualism vs. holism, materialism vs. idealism, implicit vs. explicit), Rosa works with a complex topology that is multidimensional and immune, at least at the metatheoretical level, to the reductionisms of orthodox critical theories in the Weberian-Marxist tradition (Vandenberghe 1997–1998). It should be understood that social change can simultaneously occur at all levels of self-interpretation – it can move up from individuals to society (individualism) or percolate from society to individuals (holism). Similarly, ideational change can transform social practices (idealism) as much as transformations of feeling structures can instigate cultural change (materialism). When the strains between the explicit and the implicit dimensions at the individual and the collective level become too strong, a whole series of crises and pathologies may lead individuals and societies to the brink. This focus on social, cultural and personal change will be taken up again later in his habilitation thesis on acceleration.

### Being human 2: historical semantics

In some of the central texts of his *Philosophical Papers*, Taylor (1985a and b) has sketched out his philosophical anthropology by drawing out the connections between webs of signification, moral landscapes and a sense of self in general. The internally related concepts of “Self-Interpretation”, “Strong Evaluation” and “Articulation” form the basis of his cultural hermeneutics and his moral phenomenology of the self. Although Taylor makes a distinction between a fundamental philosophical anthropology and a more historical one, it is not difficult to see that his moral hermeneutics are nurtured by a holistic worldview that is not fully secularised. For a post-secular humanist approach that does not deny the spiritual dimension of human existence, like the one Jürgen Habermas (2019) defends in his genealogy of Western philosophy, this indebtedness to a religious tradition is not in itself problematic. But its contents need to be fully explicit and brought into language so that they can be fully rationalised, secularised and sublimated in a language that transcends (and thus also includes) other communities of faith and also beyond faith. In this spirit of religious sublimation of religious contents, I welcome Taylor’s cultural hermeneutics as an important contribution to an interpretative sociology of modernity. Without grounding in a metaphysical worldview, the holism loses its ontological flavour. It becomes properly methodological and turns into hermeneutics. As such, it is a crucial tool to uncover the collective dimension of world-making in a disenchanted world. Against hypercritical approaches that reduce action to its instrumental and strategic dimension, a cultural approach that discloses meanings, evaluations and expressions, even where and especially when actors solely seem to be driven by interests, maintains the social sciences within the remit of the human sciences. The idealistic surplus of “social imaginaries” makes intercultural comparisons possible, and it does so even better in my opinion when its spiritual ballast is minimised.

In the *Sources of the Self* (1989) and its sequel, *The Secular Age* (2007), Charles Taylor presents an anamnetic hermeneutics that wants to retrieve, articulate and actualise the intellectual traditions of the West that form the moral background of the constitution of the modern self. In broad sweep, with remarkable erudition and in analytic style, he distinguishes three historical-cultural streams that are the wellspring of modern identity: Theism, naturalism and expressivism. He does in no way deny the tensions between the different sources of identity. To the contrary. One of his central diagnostic claims is that the current malaise of modernity stems from a hegemony of the naturalist conception of the self over expressivist and theist ones. This cultural hegemony reveals itself in a naturalisation of naturalism: as it becomes the default option of identity, its normative appeal is muted, while the other visions of the place of the self in the world become subservient to a project that seeks to control the self, others and the world.

### Naturalism

The first and oldest stream, which has largely dried up or gone underground in the secular age, is theism. It assumes that God created the world and that He is the ultimate good. The believer aspires to a life beyond human flourishing that realises God’s plan on earth as in heaven. Whoever one is and whatever one wants to accomplish in life has to be oriented to God. The demise of this conception of “fullness” and “holiness” is treated at length in *The Secular Age* (Taylor 2007). In the *Sources of the Self*, theism is largely sublimated in the romantic counter current of expressive individualism. It conveys *in pianissimo* what remains of ontological holism in the modern age when the religious order has broken down, the Church has lost its monopoly of interpretation and religion has become a personal and intimate affair.

The second stream is naturalism. It is the dominant worldview since the Enlightenment and assumes that the workings of the world can be fully understood by human beings, explained by science, and controlled by technology. It values the objectivity of reason and conceives of the human being as a cold and disengaged (masculine) observer in a lonely world that is slightly threatening. The relations to the world are mainly instrumental-manipulative and strategic-utilitarian. Tracing the origins of the unencumbered, free and rational self to the writings of Descartes, Kant and Locke among others, Taylor describes the modern self as a “punctual or neutral self” (Taylor 1985a, b: 49–50)—“punctual”, because it is defined in abstraction from the moral concerns that define a self, and also neutral, because it does not only make abstraction from values, but also from affects and emotions. Later, in the spiritual sequel to the *Sources of the self*, the punctual self will reappear in the guise of a “bounded or buffered self” (Taylor 2007: 27, 37, 300).

Completely disengaged from the world, others and himself, this lonely figure objectifies, reifies and neutralises everything he encounters in the world. Between himself and the world, there is a chasm, a wall even, which separates him from his body, his fellows and the cosmos. His relations to the world are cold, unresponsive and mute, as Rosa will phrase it later. There is no resonance, only dissonance. Clearly disapproving of the naturalist worldview and its bounded self, Taylor associates it with all the blemishes of scientism, mechanism, instrumentalism, capitalism, utilitarianism, liberalism, proceduralism, atomism and individualism. At the same time, bringing his hermeneutic perspective to fruition, applying methodological holism to disclose the contours of moral individualism, he makes it clear that the emergence of this self is not just a historical aberration. Behind its mechanics, one can discern a positive image of Man as a free human being. Emancipated from religious tutelage, political bondage and economic servitude, the modern self is in control and treasures individual autonomy above interdependence. Although naturalism sees itself as an objective and neutral worldview, it speaks nevertheless from a moral position that it cannot acknowledge. It ensconces a normative vision of identity and society that is not without attraction. To be free, disengaged and in control is convincing, inspiring and moving. Hence, it comes as no surprise that it continues to orient and motivate action up till today, not just in science and business, but in all spheres of life. One could even go further and make the case with Kant, Hegel and Marx that in modernity the right to freedom is so fundamentally entrenched that any ethics and politics necessarily presupposes it (Honneth 2013). The acknowledgment of the principle of freedom does not mean that one has to stick to a negative conception of freedom or that one cannot go beyond liberalism. Rather it reminds one that in modern societies one cannot fall below it.

### Expressivism

The third stream is “Expressivism”. In frank opposition to instrumental reason, it seeks to be responsive to one’s inner voice. In France, the reveries of Rousseau are a point of departure; in Germany, the Romantic movement of the *Sturm und Drang* will continue to reverberate in the work of Herder, Goethe and Hegel (Taylor 1975: 3–50). It represents a romantic counter current to the dominant worldview of modernity. Unlike the Enlightenment view that severs the self from the world, fracturing it into oppositions between body and soul, ego and other, individual and society, Romanticism yearns for unity. It does not strive for disengagement, but for participation. Against a dead mechanical view of the world, it seeks “resonance” (Taylor 2018).

If naturalism values self-determination and freedom above anything else, expressivism prizes authenticity. The deepest aspiration of the expressive self is to become a work of art – unique and universal at the same time. In romanticism, the search for one’s inner nature finds it fulfilment in self-transcendence. When the subject feels most intimately connected to nature, others and the universe, when everything resonates in the soul, the subject supposedly has found and realised its authentic or alethic self.

Once Hartmut Rosa will have reformulated his social theory in relational terms and connected it to critical theory, Taylor’s opposition between naturalism and expressivism will reappear as we will see as an opposition between two modes of being-in-the -world, namely alienation (which disconnects the self from the world) and resonance (which reconnects it to the world) or, in more existential lingo, *Geworfenheit* (being thrown) and *Geborgenheit* (being held).

### Autonomy and authenticity

Charles Taylor is a progressive Catholic philosopher with strong Franciscan sympathies. His ultimate position seems to be that the three cultural streams of theism, naturalism and expressivism continue to be available as moral resources in the secular age. Naturalism is the dominant paradigm of the modern age; expressivism/romanticism the counter paradigm. Although theism has gone underground, it still secretly nurtures the other two sources. Taylor’s reconstructive genealogy of the moral landscapes of modernity acknowledges that autonomy is the hegemonic hypergood behind the instrumental actions, but he hopes that through articulation of the moral worldviews of theism and expressivism, the hypergoods of plenitude (theism) and authenticity (expressivism) can nevertheless be partially retrieved so as to inspire personal development and collective action.

While the values of freedom and efficacy are most fully institutionalised in the autonomous subsystems of society (the economy, the administration, technology and law administration and law), the value of authenticity is central to the humanities, the arts and education. It has been captured, though, by mass media and commercial culture or retreated to the private sphere. The contemporary infatuation with “selfies” shows that the desire to be authentic is increasingly framed by commercialism. The “deep identity” of the punctual ego colonises the “superficial identity” of the singular self with the result that the quest for authenticity degenerates into the heteronomy of capitalist alienation. Convinced that the formation of identity can only succeed if there is a culture and a community that actively sustains it, Taylor proposes a “communitarian agenda” (Rosa 1998: 433–470). It encompasses a remoralisation of the public sphere (“the common good” [*Gemeinwohl*]) and a politicisation of the private sphere ("the public spirit” [*Gemeinsinn*]). When the community consciously defines the “common good” and democratically decides to collectively pursue a “common project” that expresses, nurtures and sustains its identity, the social and political preconditions of a “good, beautiful and full life” can possibly be satisfied. In theory, the communitarian agenda is sound, but as the current worldwide backlash painfully demonstrates, in practice, the proposal to remoralise public life and politicise private life may countenance the ascent of moral majoritarianism and political populism as a reaction of resentment against identity politics and “wokism”. I will return to these issues at the end of the article.

Both Taylor and Rosa are caught between the multiple strands of the modern imaginary. They deplore the hegemony of naturalism in all its variants; yet, as good Hegelians, they cannot simply give up the abstract morality of freedom to the ethics of authenticity. Although they tend to oppose morality to ethics, the just to the good, self-determination to self-realisation, autonomy to authenticity, and control to contemplation, they know that the streams have mingled, mutually influenced and transformed each other over the last centuries without ever arriving at a stable equilibrium. Like Max Weber, they accept value pluralism and counsel endurance when it comes to axiological conflicts in modernity (1998: 361–387). They know no return to the ancient hierarchical order of yore is possible, yet they still aspire to some kind of integration in the greater whole. In their work, the romantic aspiration to a meaningful order is sublimated in the methodological holism of cultural hermeneutics and conjoined with a communitarian defence of moral individualism and a critique of utilitarianism.

The moral inquiry into the conditions of self-realisation and self-actualisation shows that they operate within the modern horizon of individualism; yet, they also understand that the individual partakes of a community of opposing meanings and values that constitutes it. Autonomy and authenticity are in tension with each other (Ferrara 1994); but they also complement each other. As entangled hierarchies of subordination they may be flipped over, so that one may criticise one moral paradigm in the name of the other. It is only when one master frame becomes dominant to the point of assimilating and suppressing the other that it becomes properly ideological and pathological. The accumulation of social pathologies and existential crises signal that the predominance of instrumental rationality over value rationality in modern societies is not sustainable. The loss of community, meaning and resonance are so acute that they threaten the very freedom that the “project of modernity” advanced against the traditional order.

In his theory of social acceleration, which is the subject of his next book, Rosa will reformulate the dialectic of the Enlightenment (Horkheimer and Adorno 1987) and the legitimation crisis of late capitalism (Habermas 1973) with the help of Charles Taylor. He will argue that the modern imperative to control has brought into existence a system that is out of control. The hegemony of naturalism over expressivism is almost total and has spawned autonomous systems that turn ever faster and undermine personal autonomy. The system spins on itself and empties life of its meaning. Following the diagnostic theories of post-modernity, he will conclude that modernity has failed. Reason is no longer seen as being part of the solution. It is part and parcel of the problem of modernity.

## A social theory of acceleration

Hartmut Rosa’s interest in the phenomenon of acceleration can be traced back to the year he spent as a post-doc at the New School of Social Research in New York in 2001–2002. His interest in the sociology of modern time is evidenced in the reader on acceleration and power he edited with William Scheuerman (Rosa and Scheuerman 2009). It contains texts by Georg Simmel, John Dewey, Carl Schmitt and Reinhardt Koselleck among others. In 2004, he obtained his Habilitation in sociology and political science at the university of Jena with a celebrated book on social acceleration. *Beschleunigung* (translated in English as *Social Acceleration*) is a thorough investigation of the cultural transformations of the temporal structures of modernity. Published in 2005, this bestseller (more than 10 print runs since) impresses by its scope and ambition, the range and variety of its theoretical sources, the complexity of its composition and the clarity of its lines of argumentation (handily summarised in Rosa 2003 and 2012: 185–223).

The eponymous book presupposes the cultural hermeneutics of his doctoral dissertation, but it is also much more oriented towards the social sciences. The central figures in the theoretical landscape are no longer moral and political philosophers (Taylor, MacIntyre, Walzer, Habermas and Honneth). Classical sociologists (Marx, Weber, Durkheim, but above all Simmel), theorists of late modernity (Giddens, Beck, Bauman, Castells, Bauman) or post-modernity (Baudrillard, Virilio, Sloterdijk, Harvey, Jameson) now occupy the midlands. The conceptual shift from “moral spaces” to “timescapes” coincides with the discovery of Reinhardt Koselleck’s oeuvre. The father of German *Begriffsgeschichte,* who didn’t even figure in the bibliography of his monograph on Charles Taylor and who put the theme of historical acceleration on the map, is now—and justifiably so—the most cited author.

Taylor’s moral critique of the “malaise of modernity” is still present, though, albeit in muted form. In the background of his analysis of late modernity, one senses that Rosa is fleshing out Taylor’s (1985b: 248–288) critique of the growth paradigm of the affluent society. Taylor wonders why the majority of the population accepts the neo-corporatist compromise and is willing to exchange “alienated labour in return for consumer affluence”. His answer is that industrial-capitalist societies satisfy the aspirations of autonomy and efficacy. The consumerist definition of the “good life” as “continuous escalation in living standards” (id., 280) explains “the fixation on brute quantitative growth” (ibid.). So, if “the machine must run on”, it is because growth satisfies the aspirations to a good life as a life of plenty.

The Canadian philosopher announces at the same time an impending “legitimation crisis” of capitalist productivism-cum-consumerism. The tendency towards concentration, centralisation and expansion “inexorably destroys smaller communities” (id. 250); the increased mobility of people breaks down “long-standing ties between people” (ibid.). The moral protest against endless growth is fuelled by neo-platonic critiques of pleonexy (greed), romantic appeals to return to nature and Marxian critiques of irrationality. Together, they configure a revolt of authenticity against the dominant narrative of modernity. In Rosa’s hands, Taylor’s moral critique of the “runaway machine” will be worked out in a full-scale sociological critique of the logic of acceleration, accumulation and growth that slowly leads modern societies to self-destruction.

### A social and diagnostic theory of temporal structures

In comparison with his first book, *Acceleration*, his second book, is less concerned with the metatheoretical foundations of the human sciences. The study of high-speed societies endeavours to reconceptualise some central problems of sociology and diagnose some of the central problems of modernity. If we follow Andreas Reckwitz’s (2021: 25–44) distinction between “social theory” (*Sozialtheorie*) and “theory of society” (*Gesellschaftstheorie*), the former dealing with the general problem of synthesis (What is society?), dynamis (What are the driving forces of social change?) and praxis (How can actors deliberately change society?) (Rosa and Schulz, 2023), the latter with the structural characteristics of modern and contemporary societies, we can more clearly situate Rosa’s chronosophical reflections at the intersection of social theory and the theory of society. By focusing more intently on the experience of time and the changing temporal structures in modernity, he’s able to significantly renew Anthony Giddens’s theory of structuration, connecting everyday life via culture to systemic change, while presenting an innovative critical analysis of accelerating social, cultural and personal change in late modernity. A social theory of time, set up to capture the “signature of the times” is what Rosa accomplishes in his impressive diagnosis of the present: “A *Zeitdiagnose* in the most literal sense of the term” (2005: 38).

For Rosa, time is everything. It is the focal point of an intricate conceptual arc that aims to span a triple gap in an encompassing social theory of acceleration with totalising, normative and critical intent: Between agency and structure (or “life-world” and “system”); between classic and late modernity (or “first” and “second modernity”); and also between social theory and moral philosophy (analysis and critique), which will be parsed in the third phase of his work. The following quotation clearly spells out the theoretical ambitions of his sociology of temporal structures: “If temporal patterns and perspectives represent the paradigmatic site for the mediation of structure and culture, of system and actor perspectives, and therefore also of systemic necessities and normative expectations, so this immediately suggests that they disclose a privileged point of entry for the social-scientific analysis of the entire cultural and structural formation of an age” (2005: 38).

### Synchronisation and desynchronisation

Since Fernand Braudel’s (1958) celebrated distinction in the *Annales* between the three temporalities of events (“battles”), conjunctures (“cycles”) and structures (“civilisations”), the question has arisen how the subjective experience of time can be connected to large-scale societal change. Rosa answers by pointing to the changes of temporal structures and horizons over time that sway social systems, social actors and everyday life. Drawing on the sociology of time, he shows that time structures are socially constructed, historically variable, cultural representations that modulate the self-interpretations at the individual, collective and historical levels of societies. In modern times, the necessity to coordinate and integrate individual actions and systemic operations has given temporal structures a pivotal position in the reproduction and transformation of industrial-capitalist societies. The imperatives of synchronisation regulate all spheres of life (family, education, work, leisure, etc.) and simultaneously transform practices, individuals and societies as a whole. As a result the integration between the levels of daily time (*Alltagszeit*), biographical time (*Lebenszeit*) and historical time (*Weltzeit*) becomes ever more tight.

The systemic need to synchronise activities across time and space is accompanied by multiple desynchronisations between the different time-scales (daily experience, life course, history). At the level of practices, the alteration of the routines of everyday life (reading newspapers, checking e-mail, going to the gym, etc.) has significantly changed the course of the day. 24/7, day in day out, we’re packing ever more activities in the span of a single day, week or year (Crary 2014). Even during the night, we’re increasingly restless and agitated, suffering from a whole series of sleeping disorders, like apnoea, insomnia, narcolepsy, restless legs syndrome, delayed and advanced sleep phases and even fatal familial insomnia (Wolf-Meyer 2012). At the level of the individual’s life course, biographies have become reflexive and subject to planning, yet also more contingent, as the theory of “reflexive individualisation” (Beck and Beck 1994) has pointed out. At the level of society, time itself seems to have accelerated, leading up, objectively, to systemic and social disintegration and, subjectively, to a generalised disorientation.

Rosa dramatizes Reinhart Koselleck’s (2000: 150–176) well-known thesis of the acceleration of history since the eighteenth century and, transposing it to sociology, he introduces the logic of social acceleration as the driving principle of modernity. Whether one thinks modernisation, first and foremost, as a process of division of labour and functional differentiation (Durkheim), commodification and exploitation of nature (Marx), formal rationalisation and bureaucratisation (Weber) or individualisation (Simmel), underneath of these master processes, one can discern an anonymous logic of dynamic stabilisation/destabilisation that transforms simultaneously the structure, nature, culture and personality of modern social systems, as Rosa (2005: 89–111, 428–459) says in a rather loose application of Talcott Parsons’ AGIL-model.

Rosa complements Parsons’ functionalist version of evolution, which is indeed upbeat, with a counterpunctual analysis of the “dark side” of modernisation that highlights its paradoxical reversals. As social change speeds up, functional differentiation turns into structural disintegration, the exploitation of nature clears the way to its destruction, the rationalisation of culture induces disenchantment, and individualisation is accompanied by massification. Capping the whole process, speed-up itself leads to the “fossilisation” (Weber), “crystallisation” (Gehlen) and “reification” (Adorno) of history (Vandenberghe 1997–1998, vol. I).

In a co-authored piece, Rosa and two of his colleagues and sparring partners from Jena, define “dynamic stabilisation” as a paradoxical mode of metastable reproduction: “Modern societies appear extremely dynamic with respect to their high rate of growth, innovation and change, on the one hand, and quite stable in terms of their basic socioeconomic structures, on the other hand” (Rosa et al. 2017: 54). Like in Gramsci’s “passive revolution” (Gramsci 1988), everything changes to stay the same.

Once again, the dialectic of the Enlightenment is at play in the paralysing process of social change. Acceleration undermines the principles and hypergoods (freedom, autonomy, efficacy) in whose name the growth machine was set in motion. The obsession with the control of nature, others and the self has turned into self, social and systemic domination by large-scale processes that are themselves autonomous and out of control. History loses its direction and the dialectic comes to a “frenetic standstill” (Virilio, *apud* Rosa 2005: 55, 460 sq.). When the future is blocked and the return to the past is impossible, time is “detemporalised”. Like in a postmodern version of Zeno’s paradoxes, the arrow of time becomes motionless. As it advances in high speed towards the future, the present stretches to infinity. In the twenty-first century, the dynamism and “futurism” of the early twentieth century slowly gives way to the immobility of today’s “presentism” (Hartog 2003).

### The acceleration of history

The prophetic thesis of the acceleration of history—and its counterpoint: reification – is parsed out, specified, and even operationalized so as to be measurable, in a systematic theory of societal morphogenesis that distinguishes: 1) technological acceleration, 2) the acceleration of social change and 3) the acceleration of the pace of life as three analytically distinct dynamics that are empirically intertwined in a Juggernaut of speed (1985: 159–240). As we’ll see in the next section, each form of acceleration is propelled forward by a distinct “motor”: economic (capitalism), organisational (competition), structural (functional differentiation) and cultural (secularisation).

(1) Technological acceleration is an intentional, goal-directed, teleological process that seeks to speed up the transportation, communication, production and circulation of goods, services and people across time and space. Techno-scientific “velorutions” bring about a new “spatio-temporal regime” that fundamentally changes our mode of being-in the world and, by implication, also our human identity. The modification of our relation to time and space also changes our relation to people and things. Dislocated and disconnected from stable symbolic and moral frameworks that endow our life with meaning and value, people and things become inert, devoid of meaning, while common places are transformed into anonymous spaces (Augé’s “non-places”) and stretches of time are disaggregated into digital flows (Castell’s “timeless time”).

In his Ph. D., Rosa had already denounced the proliferation of meaningless “non-places”, “non-times”, “non-persons” and “non-things” to which no one is attached and that do not offer any possibility for identification as a symptom of the “expressive poverty” (1998: 406–413) of modernity. In his later work, he will amplify his critique of a mute, cold and lifeless world in a phenomenology of the “relation of non-relation” in which an alienated self perceives the world increasingly as a “point of aggression” (2018a: 10).

(2) Unlike technical acceleration, which is conceived as a change within society, the acceleration of social change in post-traditional societies is thought of as an acceleration of society itself. It leads to a generalised morphogenesis. The scientific, industrial and political revolutions of modernity significantly accelerated social change and propelled the world into the vortex of “modern times” (in German, *Neuzeit* means literally “new time”). The accelerating rates of technological, social and cultural innovation, celebrated in communist, modernist and accelerationist manifestos, upended the patterns of habitual action. Thrown into the maelstrom of continuous change, hallowed traditions were quickly rendered obsolete. Continuous adaptation to ever-changing conditions was exhilarating, but the frenzy also caused disorientation, confusion and a dizzying sense that “all that is sold melts into air” (Berman 1982).

As social change intensified, the sense of temporal continuity was lost. This was beautifully captured in Koselleck’s (1979: 349–375) celebrated categorical distinction between the “space of experience” (*Erfahrungsraum*) and the “horizon of expectations” (*Erwartungshorizont*). Eventually, the gap was widened to the point of becoming a genuine “rupture” of temporal continuity. In sober terms, Koselleck (1979: 336) draws attention to the ensuing experience of temporal discontinuity: “The gap between past experience and future expectation widens, the difference between past and future grows, so that lived time is experienced as a rupture, as a time of transition, in which something new and unpredictable always emerges.”

Social acceleration dramatically alters the “regimes of historicity” (Hartog 2003) that articulate the relations between the past, the present and the future. It also ruptures the continuity of the timeline that interconnects generations. In traditional societies, time is almost stationary and doesn’t change much from one generation to the next one. In early modernity, the topology of time is remodelled. As stationary societies became dynamic and future-oriented, time became more linear and cumulative. From the eighteenth century onwards, social change accelerated within a generation so that different times could coexist in a same time span. The “non-simultaneity of the simultaneous”, in Ernst Bloch’s felicitous formulation, implies coexistence of different temporalities, but also confrontation between different generations. When father and son or daughter no longer share the same “space of experiences” and “horizon of expectations”, temporal continuity is upended. The present contracts, the past becomes obsolete, the future uncertain. In the late twentieth century, history accelerated once again. The pace of change is now intragenerational. One has the unpleasant feeling that one needs to permanently retool and reschool to simply stay update. The past is gone and no longer serves as guide to the future, which becomes, as Koselleck says, “unpredictable”.

(3) The acceleration of the pace of life was at the centre of Georg Simmel’s modernist philosophy. In the last chapter of his *Philosophy of Money* (Simmel 1989), he presented a coherent vision of a world in dissolution and highlighted the speeding up of the rhythms of daily life in the commercial metropolises (Berlin, Paris, London, New York) of the first globalisation. Empirical research on time-use and time-budgeting suggests that since then time has become an even scarcer resource. Objectively, we are doing ever more things in less time; subjectively, however, we feel under constant pressure and are stressed out by contracting deadlines and expanding to-do lists. The demands for “degrowth”, “unhastening” and “oases of decelaration” (slow science, slow food, slow sex, etc.) are a reaction to social acceleration and derive from a felt need to wind down.

### The motors of acceleration

The examination of the three dimensions of social acceleration reveals a feedback system in which technological innovations (from the steam engine to the car and the laptop) almost inevitable bring about increased rates of social change, which cause a speed-up of everyday life, which, in turn, spur actors to search for technological time-saving devices. Technological changes bring about changes in economic organisations, communication structures, political institutions, moral cultures, social practices and self-perceptions. In the information age, the Internet, social media, mobile phones have definitely changed the economy, politics, culture, education, subjectivity, etc.

Rosa (2005: 256–310) discerns four structural drivers of acceleration (three external ones and one internal one): 1) capitalist accumulation (the “economic motor”), 2) functional differentiation (the “systemic motor”), 3) competition (the “organisational motor”) and 4) the idea of an intensive life (the “cultural motor”).

(1) With Marx, he shows that the accumulation of capital has brought into existence an economy that is hooked to growth. The dynamisation of production, distribution and consumption of goods and services is explained by the profit motive. Focusing on consumption, he notes that publicity incites us to go shopping and to buy ever more products that we no longer consume: “Who shops doesn’t consume” (2009c: 276), as he pithily puts it. We don’t have time anymore to read all the books we bought and to watch all new series on one of the streaming services we subscribe to.

(2) With Niklas Luhmann, he insists that the necessity to reduce the complexity of social systems with regard to their environment compels the functionally differentiated subsystems of modernity (the economy, law, science, etc.) to develop programmes with specialized media and codes of communication that increase the options and possibilities in the face of an ocean of contingency. The reduction of complexity is accomplished through a temporalisation of the elementary operations of the system that accelerates the system as a whole.

(3) In a later text, Rosa (2012: 324–353, see also 2009: 44–49) adds competition within organisations as a turbo on the economic engine of acceleration. The principle of competition allocates personnel and resources on the basis of merit and performance. It originated in economic organisations, but has now spread to all types of organisations (science, politics, sports, culture, etc.) and produced a type of subjectivity that is always looking to augment its chances on the market. In order to remain competitive, individuals are forced to augment their options and opportunities and follow von Foerster’s categorical imperative: “Act at all times to increase the number of options and possible connections you have” (2012: 349).

(4) The motorisation of the capitalist economy, social systems and organisations is sustained by the cultural idea that a successful life is an intensive life, full of action and experiences. As life is short and unpredictable, one should continuously experiment, sample strong experiences and chase different sensations so as to pack as many lives into a single life. The aestheticisation of life and the idea that one can realise oneself through an intensification of experiences reveal to what extent the idea of authenticity is framed by the idea of autonomy. Post-modern expressivism is not opposed to cultural capitalism; colonised by it, it expresses it and instigates the subjects to continuously renew their appearances, publicise their life (on TikTok, for example) and become who they really are.

### The reification of temporal structures

The synergies between the processes of capitalist accumulation, functional specialisation, performative competition and personal self-realisation have created a self-perpetuating, unstoppable and almost mythical logic of incessant growth, innovation and escalation that permeates all spheres of life and imposes itself on everybody, independently of their will. The idea that one should always maintain one’s options open and expand the reaches of one’s action has transformed life into a perpetual rat race. The Darwinian struggle of the fittest culminates in the “survival of the fastest” (Rosa and Scheuerman 2009: 8). In order not to fall back, professionally, one has to speed up life, self-optimise and run from one project to the next one. Without stability, the new spirit of capitalism feeds on insecurity and breeds continuous innovation (Boltanski and Chiapello 1999).

Temporal practices that once served an adaptive function and made sense in the transition to modernity have crystallised into inert temporal structures that operate “behind the back of the actors”. Alienated from the values and ends that initiated them, temporal practices are reified into quasi-autonomous, pseudo-natural processes that impose their “objective constraints” (*Sachzwänge*) on everyone and everything without consideration. They normatively regulate our practices, from without and from within. They do so without explicitly formulating norms and without our consent. From without, social acceleration is imposed by the facticity of an autonomous and anonymous logic of social systems that are programmed to turn ever faster.

The case of academia with its demands of internationalisation and impact, academic entrepreneurialism and independent fund-raising, its obsession with excellence, audits and metrics, the substitution of tenure-track positions for short-term contracts, etc. (Pels 2003) is the one we all know best from experience. Whether we want it or not, production and consumption, communication and transport are always speeding up. Objectively, we’re always doing more in less time (more admin, more research, more grants); subjectively, we feel nothing really gets done. We’re getting exhausted and burned-out by ever-increasing demands and a lack of time to do what we really want to do: spend time with the family, read or write a book, go on holiday or contemplate the sky.

From the perspective of Charles Taylor’s critical hermeneutics, the reification of temporal structures, the alienation of forms of life and the production of an instrumental way of being in the world can be understood as the result of the dominance of the value of autonomy in modernity. The insistence on disengaged reason, individual freedom, instrumental efficacy and affective neutrality has ensued in an inhabitable world that undermines the very subjects it was supposed to serve. The pursuit of individual autonomy has led via unintended, but inevitable consequences, to the emergence of quasi-autonomous systems that undermine the community, strip life of its meaning, and threaten individual freedom and collective self-determination.

### The failure of the project of modernity

Meanwhile the social processes (exploitation of nature, rationalisation of culture, structural differentiation and individualisation) that were set in motion with the advent of modernity have been swept up once again by the twin processes of acceleration (through time) and globalisation (through space). The classical analyses by Marx, Weber, Durkheim and Simmel of the “first surge” of global acceleration at the turn of the twentieth century (1880–1920) have to be updated with an analysis of the “second surge” (1989–2008) of accelerated globalisation at the turn of the twenty-first century. To understand the new challenges of the “second modernity”, Ulrich Beck and Hartmut Rosa (2014) have co-authored a piece in which they combine their respective perspectives in a theory of “reflexive dynamisation” (a contraction of Beck’s “reflexive modernisation” and Rosa’s “dynamic stabilisation”). Accelerated globalisation has interconnected societies, cultures and persons in a global “community of fate”. It has to face simultaneously the risks of ecological breakdown (global warming), economic meltdown (the great recession), political upheaval (populism), pandemics (Covid-19) and geopolitical strains (wars). Beyond a certain threshold, which, as a good German, Rosa situates around 1989, the accumulation of global risks reached a tipping point. Crises proliferate, pathologies maturate, and, slowly but surely, highly developed societies start to disintegrate. Swept up by the autonomous logic of hyperacceleration, nature, culture, structure and personalities start to fall apart.

The promises of both personal and collective autonomy have not been realised. The intertwined processes of economic accumulation through “appropriation”, technological innovation through “acceleration” and political regulation through “activation” (Dörre et al. 2009; see also Rosa et al. 2017) have led to a generalised disorientation, both at the individual and the collective level.

In late modernity (though at this stage, one might as well say “postmodernity”), processes of “reflexive individualisation” have dissolved traditional identities. Set free from tradition, individuals are obliged to plan their own life and craft their own biographies. The obligation to be free and constantly choose who one wants to be has, however, introduced a good deal of contingency in the life course. The pressure to continuously adapt to the changing circumstances has undercut the possibility of projecting oneself into the future and to engage in long-term commitments. As a result, identities have become “situational” (2005: 352–390 and 2012: 224–265). Unable to foresee the future, subjects start drifting.

At the collective level, the loss of historicity and the exhaustion of utopian energies have led to the impossibility to democratically steer society and to plan its development over time. Politics has also become “situational” and “reactive” (2005: 391–427). Too slow to deal with fast systems, like science (Big Science), technology (Big Technology) and the capitalist economy (Big Business), it reacts to immediate pressures, “muddles through”, and abandons long-term planning. The desynchronisation between different subsystems puts the political system in a bind. While the shortening of its temporal horizon makes time resources more scarce, the long-term effects of scientific, technological and economic change widen its temporal horizon. Not much can be done, however. It is difficult to accelerate politics. It takes time to deliberate and decide about complex and urgent issues, so those are handed over to non-democratic and non-majoritarian institutions (like central banks, constitutional courts and international organisations).

As the promises of self-determination have not been realised, Rosa (2005: 451–459) is tempted by postmodern diagnoses and concludes that the “project of modernity” has failed. Everything changes, but the movement leads nowhere. As a result of hyperacceleration, history and politics have arrived at their end. The “eternal return” of the same has finally arrived. That does not mean that nothing happens or that nothing can be done. Bereft of proper steering mechanisms, changes have lost their direction, however. Societies are spinning like tops and individuals are drifting, while ecological, geopolitical, economic, political and social crises accumulate.

## A critical theory of alienation

### Diagnosis of the times

*Social acceleration* was written at the intersection of social theory (*Sozialtheorie*), the theory of society (*Gesellschaftstheorie*) and the diagnosis of the times (*Zeitdiagnose*). In a social theoretical analysis that skilfully interweaves the time scales of everyday life, the life course and world history, it uncovered the logic of “dynamic stabilisation” as the dominant socio-logic of modern times. From the beginning, Rosa’s totalising analysis of the “acceleration of social acceleration” was inseparable from a critical diagnosis of the present. It presented, literally, as we have seen, a diagnosis of the times.

In German, *Zeitdiagnosen* constitute a well-defined genre within the theory of society. From Tönnies, Weber and Simmel (Berlan 2012) via Mannheim, Gehlen and Schelsky to Habermas, Beck and Luhmann (Lichtblau 1995), German social theorists have painted their societies in broad-brush, coining snappy concepts (like the “risk society”, the “high-speed society” or even the “diagnostic society”), to indicate developmental tendencies, intimate epochal ruptures and warn of social crises and social pathologies.

Interpretations of the “signature of the age” typically aim at two different, but interrelated publics: The academic community of peers on the one hand and the enlightened public on the other hand (Nassehi 2001). Written by social theorists, they transcend academia and use the form of the essay to reach the public sphere and inform public debates about the future of society. Diagnoses of the times are “self-interpretations” (or “self-descriptions”, as Luhmannians like Armin Nassehi would say) by which a society that is going through significant upheavals in all spheres of life observes itself. These self-interpretations are most often associated to “strong evaluations” by which various scenarios of development are judged, evaluated and ranked according to their desirability. These social self-interpretations and -evaluations are not just self-observations, however; they are interventions in society that have the potential to actively contribute to the constitution of society.

Following once again the social philosophy of Charles Taylor, Rosa (2021: 165–167) considers his diagnosis of the acceleration society as a “best account” of the actual situation. It incorporates empirical research from various disciplines in a totalising vision of the present situation. It describes, interprets, explains and judges social systems, processes and practices and brings all evidence together in a synthetic narrative about the human condition in late modernity. A best account of the present situation is above all intended to be enlightening. It articulates the self-understandings of a society, expresses its aspirations, but also its fears of disintegration and disorientation. In this “double hermeneutics” (Giddens 1982: 1–17) between social science and common sense, there is a continuous interplay between the scientific diagnosis of society and the collective self-analysis in the public sphere by society. The scientific discourses feed on collective self-understandings and bring them via the media to greater clarity, so that the community can take the decisions it needs to face turbulent times and come to terms with its aspirations and fears.

### Critical theory and moral philosophy

A theory of society typically combines social theory and social diagnostics (Rosa et al. 2020:11–16). A theory of society becomes a critical theory when it explicitly formulates the normative criteria of its diagnosis and develops a systematic critique of social injustices and social pathologies. In the tradition of the Frankfurt School, social theory is inseparable from social, moral and political philosophy. Whereas the diagnosis of the present presupposes criteria that allow one to distinguish the normal from the pathological, the justification of these criteria remains, however, a properly philosophical task. Within the second Frankfurt School, two different strategies of normative foundation can be distinguished (Stahl 2013). A more procedural version that harks back to Kant seeks to ground its moral judgments in (quasi-)transcendental fashion in the formal conditions that make a rational consensus possible. This is the strategy of Karl-Otto Apel, Jürgen Habermas and Rainer Forst. The other strategy, followed by Axel Honneth, Charles Taylor and Hartmut Rosa, takes a Hegelian route. It denies that one can arrive at universal truths that are transhistorical or extra-social. It argues instead that one should judge a society in its own terms and that critique should therefore be reconstructive and immanent.

The distinction between transcendent and immanent critique does not fully coincide with the distinction between liberalism and communitarianism in contemporary moral philosophy (Forst 1993), but it is related to it. Communitarian theories contest the universality and ethical neutrality of liberal theories of justice, like Rawls’s and Habermas’s. Although they pretend to be independent from particular conceptions of the good, their contractual vision of justice as fairness and of the individual as an “unencumbered” (Sandel) or “punctual” (Taylor) self betrays that they express and embody a particular vision of the good life that is typically modern and typically Western. In opposition to theories of justice that invent ideal societies and procedural republics without discrimination in which goods and rights would be distributed evenly and fairly, communitarian theories of the good tend to insist that identities are shaped by different kinds of constitutive communities. As these communities are constitutive of individual and collective identities, communitarians assume an obligation to support the communities that sustain these identities and give meaning to the life of their members.

From the point of view of critical theory that joins Nietzsche’s critique of culture with a Left-Hegelian critique of alienation, forms of life that do not offer the social conditions for self-realisation and self-actualisation and systematically thwart human flourishing may be considered “pathological” (Honneth 2000: 11–69). Whereas theories of justice press for the redress of inequalities of basic goods through politics of redistribution, theories of the good life denounce forms of life in which social pathologies, like alienation and reification, anomie and disenchantment, depression and panic attacks, abound as misdevelopments. Anchored in the social suffering of the masses, young Hegelians ground the “emancipatory interest” in a desire to transcend society that already exists in society. Ideally, a critical social theory articulates normative visions of individual and collective self-determination (autonomy) and self-realisation (authenticity) and uses those visions of “the good life with and for others in a just society” (Ricoeur 1990: 199–236) to evaluate, judge and criticise societies that do not live up to their standards.

### From acceleration to alienation

In a series of intermediary texts between *Social Acceleration* (2005) and *Resonance* (2016a), Hartmut Rosa (2009a, 2009b, 2010) resumes the discussion with moral and political philosophy of Charles Taylor that had receded somewhat to the background. He places himself explicitly in the tradition of the Frankfurt School. At the same time as he takes his distances from the second generation of the Frankfurt School (Habermas), he tries to join the first (Adorno, Horkheimer, Marcuse) and the third generation (Honneth) in a radical, but slightly conservative critique of modernity that explicitly draws on the communitarianism of Charles Taylor, Michael Walzer and Alisdair MacIntyre.

With Michael Walzer and Charles Taylor, the German sociologist assumes that social critique is strictly immanent, interpretative and reconstructive. “The appropriate standards of sociological enlightenment and social criticism come from society itself” (2009b: 28). The self-interpretations and self-evaluations of a given society do not only supply the critic with the language, the norms and the values in the name of which social criticism is proffered; they also help the analyst to translate common complaints of society into a systematic social diagnosis and critique (2012: 104–147).

Thanks to an “articulation” of existing moralities into a coherent self-image, which holds up as it were a mirror to society in which it can see itself, the critic can contribute to the public discussion by making its self-understandings explicit. This can facilitate the diagnosis of tensions, if not outright conflicts and contradictions between its explicit and implicit, ideal and real, normative and empirical self-interpretations. On the basis of such a diagnosis of tensions between the normative constitution of a society and its existing institutions, various crises (e.g. ideological crises, legitimation crises, identity crises) and social pathologies (reification, alienation, anomie) can then be identified, analysed, diagnosed and, possibly, also cured.

Now that social critique has found its grounding in the normative visions of the common good that a given society espouses, even and especially when it deviates from its ideal course, the chronosophical diagnosis of acceleration and its crises of desynchronisation can be complemented with a normative critique of the social pathologies of modernity. In the Left-Hegelian tradition of the Frankfurt School, those pathologies are typically interpreted as pathologies of reason (Habermas 1988; Honneth 2007; see also Gros, 2019). The reversal of rationalisation into reification is understood as the result of an alienation of the “objective spirit” (Honneth, 2005). Instead of realising its promises through a release of its transformative potential in history, substantive reason (*Vernunft*) shrinks into formal reason (*Verstand*) and becomes instrumental and utilitarian.

If one thinks through the reification or the alienated autonomisation of time structures from the point of view of Taylor’s critical hermeneutics, it appears as a crystallisation of cultural interpretations and moral evaluations of life courses into pseudo-natural structures. The cultural structures and personal practices behind the systems are no longer perceived as societal choices. The “objective spirit” is alienated from itself and freezes into an opaque system. When the dialectic between structure, culture and praxis stops moving, the cultural worldviews, social imaginaries and moral visions of the good life lose their causal power. The naturalist conception of nature and culture, society and subjectivity becomes entrenched and congeals into “second nature”.

The rearticulation of the self-conceptions and strong evaluations of a society aims to put the dialectic of practical reason back in movement. By showing that reification is a truncated form of rationalisation, the ideological spell of naturalism can be broken, its realisations can be incorporated and it limits overcome as the Spirit continues its adventure through history. It should be noted, however, that in his sociology of acceleration, Rosa hardly invokes the category of Reason. Following Max Weber, he speaks of rationalisation, but empties it of its emancipatory content. When he takes leave of modernity, he also says *adieu* to Reason itself.

This impression is further strengthened when he brings the old Aristotelean question of human flourishing (*eudemonia*) back on the agenda. This is a perfectly legitimate move, of course, but in Charles Taylor and even more so in Alisdair MacIntyre (1981) whom he also follows on this point, the return to virtue ethics is also a way to circumvent Kant and Hegel, revert to the Renaissance and relinquish the promises of modernity. Unlike Habermas, who tacks his own reformulation of a universalist discourse ethics to Kant’s practical reason and morality (*Moralität*) and Axel Honneth’s proposal to reactivate Hegel’s insistence on the ethical life (*Sittlichkeit*), but without its metaphysical baggage, Rosa opts for a communitarian version of neo-Aristotelianism. Instead of trying to articulate a modern synthesis of Aristotle, Kant and Hegel and explore the possibilities of a large spectrum ethics that does justice to the moral intuitions of modernity (Vandenberghe 2018 and 2021), he throws out rationality and halts somewhere on the road between Aristotle and Hegel. With the support of Taylor and MacIntyre, he reorients sociology towards the good life and human flourishing: “Whether one acknowledges it or not, the ultimate, most often implicit and even unconscious object of sociology is the question of the ‘good life’, or more precisely: the analysis of the social conditions under which a fulfilling life is possible” (2009b: 87). Or, in a more negative and sociological formulation: What are the social causes that explain that modern subjects have to lead a life that they themselves find unsatisfactory? Or, again, this time with a reference to Adorno’s (1980) *Minima Moralia*: What causes a “damaged life”?

In continuation of the diagnoses of modernity of classical sociologists and the critique of capitalism of the early Frankfurt School, the director of the Max Weber Kolleg points to the development of industrial capitalism with its escalatory logic of accumulation (growth), acceleration (speed) and globalisation (space) to explain why the ideals of individual and collective self-determination cannot be realised in modernity. The reification of social temporal structures into pseudo-natural forces that regulate social life makes them impervious to human control. In the third phase of modernity, the logic of dynamic stabilisation has become unstoppable and oppressive. Revamping the complaint about the “dictatorship of the present”, Rosa does not hesitate to qualify social acceleration as a “new form of totalitarianism” (2010: 60–63): Its temporal structures control the whole system, as well as all subsystems and all spheres of life, from the cradle to the grave, during the day as well as during the night. As we’ve seen before, he concludes that the project of modernity has failed, but now he turns his diagnosis into a violent critique of the very idea of autonomy.

Rehashing the standard communitarian complaints against Rawls’s and Habermas’s political liberalism about the privatisation of the good, he charges that their vision of autonomy is formal, empty and instrumental. Their theories are supposedly impartial, but a closer look shows them to express a vision of a successful life that supports the logic of endless growth that Taylor (1985b: 248–288) criticised in his important essay on the crisis of legitimation of the affluent society. Assuming that the good cannot be defined in substantive terms, their theories of justice focus instead on the redistribution of formal rights and basic resources. With irony, if not scorn, Rosa dubs the underlying vision of theories of justice the “triple A approach to the good life” (Rosa 2017a: 444 and 2018b): it seeks to make more and more resources “available, accessible and attainable” to the greatest number. The struggle for redistribution fuels the logic of acceleration.

Moving from the individual to the collective level, Rosa observes that the autonomisation of social systems is the systemic counterpart of the idea of individual autonomy that is at the core of the liberal vision of the good life. The “punctual” self that strives to be free, competitive and autonomous from any external determination invariably and ineluctably gives rise to systems that follow their own laws and undermine freedom (2012: 60–103). In the long run, these reified systems undermine the very freedom they were supposed to serve. They do not only affect individual self-determination. Given that technological, scientific and economic systems turn faster than the political system, collective self-determination also reaches its limits.

Theories of justice that guarantee individual rights fail to give a satisfactory account of systemically induced social pathologies. After all, one can imagine a just society in which basic goods would be evenly and fairly distributed and that still would fail to be happy: “A society can maintain complete distributive justice and still be marked by the drying up of its resources of meaning and by overwhelming, structurally caused experiences of alienation” (2009a: 28). “Alienation” (*Entfremdung*) functions here as code word for, and a particular instance of, a whole set of socially induced pathologies, like anomie, disenchantment and loneliness, that are symptoms of social suffering brought about by a loss of freedom, meaning and community in modernity. The actualisation of the old Hegelian-Marxist concept of alienation reorients critical theory away from the social critique of unequal relations of exploitation towards a more existential critique of society (Honneth 2000: 11–69, see also Haber 2007). From the point of view of a social philosophy that evaluates the conditions of the good life, reified social structures, institutions and cultural patterns that make self-actualisation of individuals and societies unlikely, if not impossible, have to be analysed, diagnosed and criticised as alienating social disorders. By systematically blocking the conditions of human flourishing, reified social structures alienate the subjects from themselves who, somehow, can no longer “live their own life”.

Inspired by Rahel Jaeggi’s (2005) existential-phenomenological reformulation of Marx’s theory of alienation, Rosa conceives of the latter as a distorted and deficient relation to the world, others and self. “The failure to establish constitutive bonds indicates that the profound distortion of the relation to the world is the central symptom and sign [signum] of alienation” (2009a: 51). In late modernity (or early postmodernity), the twin processes of globalisation and acceleration have instated a new “spatio-temporal regime” that warps the relation to the world (to places and time, to things and people, to the body and the self). It estranges the human Being (*Dasein*) from the environment (*Umwelt*), others (*Mitwelt*) and itself (*Selbstwelt)*. With a sense for drama, Rosa (2009a: 37) concludes that in industrial capitalism the “relation to the world as such and in its totality” (*Weltbeziehung ingesamt)* has become alienated. Under such circumstances, human flourishing becomes nigh impossible. Modernity is an anthropological catastrophe.

## A phenomenology of resonance and responsivity

### Good vibrations

In 2017, Rosa received his first doctorate *honoris causa* from the University for Humanistic Studies in Utrecht. Just before, *Resonance. A Sociology of World-Relation* (2016a), his second masterwork on which he had been working for almost 10 years, had been published by Suhrkamp. Although some of it had been anticipated in his inaugural lecture at the University of Jena (2012: 374–413) and in a text on Pink Floyd (Rosa, 2011), the theme of resonance quickly started to reverberate through German academia and beyond (in churches, schools, ecological movements and alternative therapies). Within academia, it has received a good, albeit not uncritical reception among social theorists, moral philosophers and theologians (Peters and Schulz 2017, Reckwitz 2017, Wils 2019, Susen 2019).

*Resonance* is a rather impressive and voluminous book of 815 pages on vibes, tunes, tonalities and attunements. With the concept of resonance, Rosa wants to “re-enchant” the world in the most literal sense of the word. Like Marcuse (1987: 241), he wants to “make the petrified world speak—to make it sing, perhaps also to dance”. Once more he has taken up a metaphor, this time not from cartography (“maps”) or kinetics (“speed”), but from acoustics. The classic demonstration of “sympathetic resonance” (which you can see  https://www.youtube.com/watch?v=aCocQa2Bcuc) involves two tuning forks: When one strikes the other, the other will start to resonate at the same frequency. The same phenomenon can also be demonstrated through the spontaneous synchronisation of two metronomes that are running in parallel (see the demonstration here https://www.youtube.com/watch?v=T58lGKREubo): Initially, they are swinging on their own frequencies; when brought together, they start to swing wildly and chaotically but, slowly, they will syntonise and, eventually, synchronise their movements in a rhythmical ballet.

Rosa used to play the keyboards in a rock band and still occasionally plays the organ in his local church in the Black Forest. Incorporating a vast literature on social theory and critical theory, phenomenology and hermeneutics, religion, history and the arts, pedagogy and psychotherapy, he has transposed a musical metaphor into a sociological symphony of a fulfilled life (*gelungenes Leben*). The book uses captions and vignettes from ordinary life (e.g. Christmas celebrations, interactions with pets, weather and mood swings) to communicate, illustrate and personalise the vibratory perspective of pathetic sociology.

The basic intuition is that the quality of the connection between the self and the world determines whether self-actualisation is possible or fails. When the relation is synergic, the world and the subject are attuned and respond to each other with love and sympathy, but also with respect for difference and alterity. At its best and its most intense, resonance is an all-encompassing “peak experience” (Maslow 1971) of full integration with the world. When the ecstatic experience of oneness-in-difference passes and settles, it gives way to a “non-ecstatic serenity, calmer happiness and the intrinsic pleasures of clear, contemplative cognition of the highest goods” (Maslow 1971: 37) that characterises a pacified existence: Everything is as it should be.

To avoid the suggestion that resonance is all “peace, love and harmony”, Rosa (2016a, b: 316–328, 484–500) underscores the dialectic of resonance and alienation: Resonance presupposes alienation. Like Georg Simmel, who considered the cultivation of interiority as a reaction to reification, he esteems that resonance entails a moment of bewilderment and surrender. Resonance is not happiness. Rather, like art, according to Stendhal it is a promise of happiness that suddenly lights up like an epiphany in the night of darkness and silence. It expresses the hope that in the midst of a hostile, silent and indifferent world something or someone will answer the call and clear the blockage that obstructs the free flow of energies and affects.

The hefty tome is constructed around a basic opposition between two modes of relations to the world: alienation/reification and resonance/responsivity. Interestingly, in an important text on Pink Floyd and Charles Taylor that offers a succinct preview of the main themes and theses of the book, Rosa (2011) traces back this stark contrast between responsive and repulsive, receptive and appropriative, erotic and thanatic relations to the world to the work of Charles Taylor. Noticing a constant oscillation in his work between an almost existentialist fear of disconnection and a humanist longing for a deep reconnection to the world, he claims that resonance is “the Archimedean point” (2011: 40) that ties the whole work of the Canadian philosopher together.

Whereas alienation undermines the possibility of an egosyntonic interchange between the self and the world, resonance makes it possible. Or, better, resonance is in itself an originary, affirmative mode of relation to the world that carries us, energises us and makes us feel whole, alive and happily connected to ourselves, others and the universe. Symmetrically inverse to alienation, resonance is its counter concept: “Resonance, says Rosa (2016a, b: 306 and 316), is the other of alienation”. When the concept of resonance is used as a measure of health and happiness (*eudemonia*), it becomes a normative concept, providing the experiential ground for an existential critique of whole forms of life that systematically block the fluid interchange between the self and its other, which is perceived as a threat that needs to be dominated, controlled or otherwise neutralised.

As we’ve seen, the concepts of acceleration and alienation were both used as totalising concepts that allowed for a diagnosis and critique of whole societies, epochs or even civilisations. The “postulate of priority of resonance over alienation” (2016a: 664) suggests that resonant relations to the world precede alienation, both ontologically and, presumably, also historically. In one sweep, Rosa has thereby un-covered an “Urform” (2016a: 435)- a basic, more primordial way of being-in-the-world. This ontology of relations not only anchors his critique of social pathologies; it also paves the way to possible therapeutics: “Another relation to the world is possible” (2016a: 56, 495 and 541).

### Resonance ethics

The disclosure of a primordial, diffuse, affective, embodied, sensual, evaluative relation to the world opens up a storey beneath the theory of communicative action (Habermas 1981). Like various other theories of intersubjectivity that have sought to soften Habermas’s rationalism with an exploration of moral sentiments (love, sympathy and care), circuits of reciprocity (recognition and gift-giving) or quasi-mystical experiences of fusion (communion, grace, ecstasy), the theory of resonance is a theory of mimesis – Adorno’s enigmatic cipher for the communicative union of difference and identity in and with nature.

In Habermas, thanks to the validity claims that are built into language, communication between subjects is geared towards understanding and reaching a consensus about state of affairs in the natural world, norms in the social world and authenticity of expressions in the subjective world. Playing out Hegel against Kant, Axel Honneth (1992) mobilised the concept of the “struggle of recognition” against Habermas’ discourse ethics and democratic politics to tap into the sentiments of indignation that are activated when subjects feel that they are not sufficiently loved, respected or valued by the others. Rather than communication, he argued, it was the non-recognition by others in the private sphere (love), by the state (law) or in civil society (solidarity) that triggered struggles of recognition that lead to moral progress and political redress of injustices.

Against both Habermas’s discourse ethics and Honneth’s ethics of recognition, resonance ethics claims that communication and recognition are not sufficient to establish synergic relations with the world (2016a: 585–595). To the contrary, vibrant relations are the motivational humus that makes communication and recognition possible in the first place. In Habermas, communication is really a meeting of minds (not of souls) in which body and emotions hardly play a role. The partners exchange arguments and test their validity, but they are not really moved or touched by the other. In Honneth, strong emotions are present, but as the struggle for recognition heats up, the chances of resonance recede. Just as there’s a basic desire for recognition, there’s a basic desire for resonance. “There can’t be a struggle for resonance, however. Resonance can neither be distributed nor allocated competitively” (2016a: 333) or, more questionably: “We cannot compete and resonate simultaneously” (2018b: 52). Moreover, both Habermas and Honneth limit the purview of their moral theories to intersubjective relations. Resonance goes deeper. It goes below language and reason, and also beyond conflict and struggle, to conceptualise the enactive relations between the subjective, the intersubjective, the interobjective and the interspiritual worlds. It incorporates the lessons of Bruno Latour’s actor-network theory, extending the reach of resonance to nature, bodies, animals and spirits to “un-earth” another, deeper, more convivial “mode of existence” (Rosa 2016b).

### Homo resonans

Rosa conceives of the human being as an embodied, self-interpreting, resonating, vibrant animal: *homo resonans,* as a contemporary variation, perhaps, of Pascal’s “thinking reed”. It assumes that human beings are basically resonance-seekers, whose deepest desire is to experience the bliss of a deep, intense and meaningful connection to everything that exists in the universe. It is only when they experience the “universal sympathy” that moves all the separate parts and connect them into an animated whole that they are fully human. Like the older philosophical anthropology of Scheler, Plessner and Gehlen, Rosa goes back to existential phenomenology and cultural hermeneutics to think the specificity of the human being as a sensing, feeling, interpreting, desiring, valuing and acting entity in the world.

In accordance with the phenomenologies of Husserl, Heidegger and Merleau-Ponty, but also drawing on the new phenomenology of “responsivity” of Bernhard Waldenfels, he anchors the relation between the self and the world in the intentionality and receptivity of the lived body (*Leib*). At the intersection of self and world, being at once a membrane, a medium and a means that interconnects both, the body is receptive and sensitive (it receives the vibrations that come from the world and is affected by them: “a ← fection”, in vectorised notation (2016a: 279, *passim*); it is also expressive and active (it reacts to the vibrations it receives and responds to affect with outgoing emotions (“e → motion”, id.). Without body, without skin and touch, without eyes, ears or mouth, without synaesthesia of the senses, no experience and no relation to the world are possible, let alone a living, vibrating, breathing and resonating relation between the self and the world.

The influence of Bernhard Waldenfels’s (2007) phenomenology of alterity and responsivity can be clearly felt in Rosa’s definition of resonance as a relation of reciprocity in which both self and the other open themselves up to each other, become attuned to each other and respond to each other “in their own voice” (2016a: 285, 298, 421, 619). Like in Mauss, the response of the other is a gift that affects and moves the recipient to respond in turn. The dialogical exchange is productive; even more, it is transformative, because when one doesn’t properly hear the other, to understand what has been said, one has to change oneself, as Taylor (1985b: 54) noted long ago. Self-transformation enhances a sense of “self-efficacy”. Resonance cannot be forced or enforced, however; it is constitutively “unavailable” and “uncontrollable” (Rosa 2018a). It comes with grace—or it doesn’t come. In any case, the synergy between the voice, the ear and a “listening heart” (Rosa 2022: 53–54) defines resonance as responsivity.

With post-Heideggerian and post-Wittgensteinian hermeneutics, Rosa assumes that relations to the world are always already symbolically mediated by language. The world is not given in its immediacy. As a life-world, it is encountered as a world that is both meaningful and valuable. The set of meanings and valuations that form the background of intentional agency may be explicit and articulated. Most often, however, they are institutionalised in collective practices and embodied in the habitus. They are, therefore, largely implicit, pre-predicative, pre-reflexive and pre-cognitive. At this point, Rosa (2016a, b: 225–235, 453–457) takes up the theory of self-interpretations and strong evaluations of his mentor and advances the claim that resonance only occurs when strong evaluations are involved. When the self encounters a sphere of resonance (like the family, religion, nature or even sports) that strongly matters to her and affects her intensely, the other starts to speak as it were. When the self responds to the other with emotion, both enter into existential communication. Now that we have all the ingredients (a ← fection, e → motion, transformative assimilation and uncontrollability, see Rosa 2018a: 37–46, 2019a: 17–20 and 2022: 58–64), we can finally define resonance as a particular way of being in the world: “By its very essence, it is an encounter with something uncontrollable, which speaks in its own voice and is experienced as a source of a strong evaluation” (2016a: 619).

### Modernity, romanticism and resonance

Resonance is an anthropological feature—a basic “capability” whose “functionings” are conditioned by social, cultural and historical circumstances. What resonates and what does not resonate varies from culture to culture. In modern Western societies, various “spheres of resonance” have been institutionalised, ritualised and materialised in a whole series of individual and collective practices that are directed towards persons, things and spiritual entities with whom one can have quasi-communicative relations. Rosa (2016a, b: 331–541) differentiates three axes of reverberation in which one can sense the echoes of the respective theories of Axel Honneth, Bruno Latour and Charles Taylor. On the “horizontal” axis of interpersonal relations, the family, friendship and politics are singled out as institutional loci that can foster intense feelings of connection with kin, colleagues and fellow citizens; on the diagonal axis of inter-objective relations, the spheres of work, school and sports are seen as domains where actors seek and find their thrills in relation to “non-reified” things (tools, musical instruments and other fetishes); finally, on the vertical axis of interspiritual relations, religion, nature, art and history are the institutional vectors that transpose human beings into higher spheres of transcendence. Although these higher spheres come last, they are, in fact, first: the Buberian “I-Thou relations” in which an Other responds in the second person to the first person constitute the “basic form of all world-relations” (2016a: 435). The mimetic relations to the numinous, which one still encounters nowadays in affect-laden encounters with charismatic persons, magic mountains, memory sites and auratic works of art, find their origin in the “sacral complex”.

Within socially institutionalised spheres of resonance, individuals have their own subjective or biographical axes of resonance. Like the personal moral maps that indicate what really matters to them, moves them and makes them move through the life course, the biographical axes of resonance arrange one’s personal interests and moral concerns in a hierarchical order of value. When specific objects (like houses, books and cars), persons (like famous philosophers, singers or actresses) and quasi-persons (like Allah, the nation or the ocean) are strongly cathected, they touch upon existential issues that makes one’s “personal axis” vibrate (the fourth axis of self-relations (2021: 249) is a later addition that remains undeveloped). Whether it is friends and family (social axis), work (material axis), ideas and ideals (spiritual axis) or one’s own self (existential axis) one prioritises, each one has to find his or her own personal axes of resonance that give meaning and direction to their life. Like in Weber’s philosophy of life, each one has indeed to “find and obey the demon that holds the strings of his life” and to make them vibrate and resonate.

With Margaret Archer (2003), we can assume that different modes of reflexivity (and thus also of internal conversations about the ranking of ultimate concerns) correspond to different modalities of self-realisation: communicative reflexives put friends and family first, autonomous reflexives dedicate their life to work, while metareflexives who seem to be moved by higher ideals seek authentic self-realisation for themselves and democratic self-determination for society. In concrete experiences of resonance, all axes swing at the same time and reverberate deep down in the soul.

In a rather revealing sentimental passage that ends with a reference to Charles Taylor, Rosa acknowledges his theory of resonance expresses a romantic longing for a world that would no longer be cold, silent and repulsive “like a desert”, but refreshing, responsive and regenerative “like an oasis”. Let me quote the passage at length, as it perfectly summarises his communitarian dream of a re-enchanted world, brimming with life and voices, that would be resonant and welcoming like a home or a homeland:

“In this way, the “Romantic model” was formative for the hope and craving for resonance, as well as for the fears of a loss of resonance, one finds in modern concepts of love and friendship; in the idea that the relations that art, music and aesthetics establish to the world have practical meaning in everyday life; in the modern experience of nature; and in certain respects even in the idea of self-realisation through work, especially where it is still oriented to the example of craftsmanship. Even more, the specifically Romantic sensitivity to and longing for resonance manifest themselves as well in the modern concept of religious experience; in the idea of education as development and not merely as training or disciplining; and in the dreams of a political community shaped by participation, solidarity and shared values, as well as by a living history. Modernity dreams of a relationship to the world that is resonant through and through, in which body and psyche, spirit and nature, individual and collective history, the individual and society overcome their divisions, encounter each other in relations of correspondence and enter into vibrant responsive relationships to each other” (2016a: 601).

If resonance is the dream of modernity, reification is the nightmare from which it tries to awaken. The possibility to distance oneself from the world and establish a cold, objective and instrumental relation to things, people and the world at large is no less a historical achievement than the capacity to mimetically and sympathetically identify with the other. In modernity, the combined logics of acceleration, competition and globalisation have, however, installed means-end rationality (*Zweckrationalität,* in Weber’s typology of action) as the default mode for acting on, and interacting with, the world. The institutionalisation of an aggressive, instrumental and appropriative world-relation in social practices, processes and autonomous systems has coincided with the relegation of more responsive, caring and sympathetic modes of world-relation to specific institutions, times and places. In the main systems of modernity (the capitalist economy, the information technology (IT) sector, interest politics, etc.) “I-It-relations” are predominant and stifle “I-Thou-relations”.

As the world axes became increasingly silent and mute, the lament over alienation grew louder and louder in literature (Kafka, Beckett, Sartre), philosophy (Schopenhauer, Nietzsche, Kierkegaard) and sociology (Marx, Weber, Simmel). Reviewing the whole tradition of critical theory, from Marx to Lukács and Horkheimer to Habermas and Honneth (Vandenberghe 1997–1998), that puts alienation and reification front and centre of their analysis and diagnosis, Rosa redescribes the tragedy of modernity as a “catastrophe of resonance” (2016a: 517–598). The world has become old and cold, unresponsive and mute, like a lunar landscape. It no longer sings or swings. And even when it does, the alienated subjects no longer respond to music. It doesn’t speak to them anymore, which is, perhaps, the clearest sign of depression.

### Resonance or reasonance?

The problem with Rosa’s sociology of the “good life” is not situated at the descriptive level, but at the normative level. From the perspective of the second and the third generation of critical theory that takes the question of normative foundations very seriously indeed, Rosa’s moral philosophy does not pass the test of universality. His spirited defence of “normative monism” (2016a: 749) leaves out essential parts of moral philosophy. Without the Kantian and Marxist heritage, human flourishing cannot be sustained (Vandenberghe 2021). The “good life for all” is a precondition for the “good life of each”. Without this basic premise, philosophy degenerates into a round-table discussion at a cosy wellness retreat between neo-Aristotelians, neo-Hegelians and neo-Heideggerians on the Left and the Right. While resonant relations may well constitute a pre-condition, as well as a consequence, of personal happiness, they do not offer a probing normative yardstick for moral evaluation and social critique. If I may coin a new word, I would say that “reasonance” is the missing term in Rosa’s ethics of resonance. The demonstration of the existence of an internal connection between responsivity and responsibility that introduces a modicum of reflexivity (“reasonance”) between the call and the answer is never provided. To test the moral acceptability of resonance, we would need something like a “norm of responsibility” or, with Bakhtin, a “norm of answerability” (Nielsen 2002). Fully articulated, it would bring back reason into the realm of resonance as an instance that transcends every actual occurrence and makes moral judgment possible.

Leaving aside occasional references to patriotism, homelands and churches, the examples that Rosa adduces to illustrate his perspective on “alienation and its other” are sympathetic, but, ultimately, too subjective. The distinction between responsive and repulsive relations, good and bad vibes, already presupposes the judgments he wants to make. That the actors themselves seemingly espouse his values does not prove, but presupposes what he wants to demonstrate. Congruence of feelings is not sufficient. It is nice, but from a moral point of view, it is not even a necessary condition. The objection that repulsive relations can come with good vibes and responsive ones with bad vibes is discarded by definitional fiat (and a little help from post-structuralism): Only cases that do not deny alterity count as genuine resonance.

All the weaknesses of communitarian ethics come to a head in Rosa’s (2019c, d) recent writings on the listening society, resonant democracy and the common good. Although he is right in taking his distances from an antagonistic conception of politics (Rancière, Laclau, Mouffe, Negri) that ontologises the struggle for power, the internal connection he tries to establish between resonant democracy and the common good is not very convincing. In the absence of a moral theory that maintains the connection to a detranscendentalised conception of reason, like Habermas’ discourse ethics or Honneth’s ethics of recognition, the communitarian appeal to shared values and visions of society is too much (too “thick” and substantial) and also too little. Hardly any commentator has failed to notice that Rosa’s vibratory politics do not pass the litmus test of reactionary resonance. Let us take a particularly grotesque, but “speaking” example: Jair Messias Bolsonaro, the president of Brazil (I live in Rio de Janeiro). His followers designate themselves as “good people” (*gente do bem*) and they refer to their hero as “the legend” (*o mito*). When they follow in their thousands the president on his motorbike (without a helmet), it is not only the engines of their vehicles that vibrate. Their heart and soul are also resonating in unison. All the axes are swinging, including the spiritual one, and, yet, it seems obvious that something is deeply amiss. It is true that Rosa (2016a, b: 370–372, 2019c: 170–178, 2019d: 209–242) makes a distinction between genuine resonance that fosters difference and “echo chambers” that repeat, retweet and amplify the same message, but once again, the distinction presupposes the moral criterion that it was supposed to demonstrate.

Resonance makes a great contribution to social theory, but it is not a moral theory. Resonance, as Dietmar Mieth (2019:184) observes, is a criterion of meaningfulness, not of morality. In the absence of a solid normative foundation that is able to categorically distinguish the voice of the masses from the voice of reason, moral judgments are reduced to personal preferences. To avoid all misunderstandings, though, I hasten to add that Rosa does not give any credence to a conservative “moral majority”, let alone to populist movements on the extreme right that want to exclude, repress, repulse and expel, if necessary through violence, whole parts of the population that do not share their hallucination of a pure community.

In accordance with some of the tenets of communitarianism, I would therefore propose a normative reduction of the remit of resonance ethics to “us”. To the extent that it already presupposes a modicum of reasonance, it marvellously expresses and captures “our” conceptions of the common good. And by “us” I mean liberal academics on the Left who still believe in the rule of law, human rights and multiculturalism and are willing to go to the streets to demand “the good life for all and every one in a just, decent and convivial society” (Convivialist International 2020).

## Conclusion: romantic anticapitalism

In this article, I have proposed a chronological and critical reconstruction of Harmut Rosas’s intellectual trajectory. In the more reconstructive parts of the piece, I have followed the temporal flow of his work and shown how throughout the four phases of his career, he has creatively worked out some of Taylor’s basic intuitions in the language of social theory. From his early work on philosophical anthropology (phase 1) via his critique of the “malaise of modernity” (phase 2) and his reconnection to critical theory (phase 3) to his latest reflections on “resonance” (2016a), “uncontrollability” (2018a) and “medio-passivity” (2019b), Taylor’s moral hermeneutics and communitarian politics have guided him through the intellectual landscape. The continuous engagement with the Canadian philosopher explains some of the main features of his social theory. His interest in a philosophical anthropology that spells out what it means to be human, both generically and historically, can be directly traced back to his Ph.D. on the social philosophy of Taylor. Without the triad of concepts of “Self-Interpretation”, “Strong Evaluation” and “Articulation” that define the basic framework of his oeuvre, one can neither understand his moral philosophy nor his theory of society. The hermeneutic imprint derives directly from the definition of human being as a “self-interpreting animal”. The insistence on “moral maps” that indicate what is good, valuable and worthwhile in life accompanies all his reflections on the “good life” and the “common good”. The importance of articulating inchoate feelings into inspiring visions of individual and collective identity explains how he understands his intellectual mission as a critical theorist who offers a “best account” of society.

Without Taylor’s genealogy of the philosophical, artistic and spiritual currents and counter currents that are at the origin of the modern conception of the self, Rosa’s critique of modernity cannot be fully understood either. In the Western tradition, three normative visions of the “full, good and beautiful life” have been spelled out: theism, naturalism and expressivism. Like Taylor, Rosa esteems that naturalism has become dominant and hegemonic. The vision of the human being as a disengaged (male) observer who is the “master and possessor of nature” has hollowed out the religious vision of Creation and crowded out the ethic-aesthetic vision of the universe. Cut off from the vital links to its environment, the triumphant self of modernity has conquered the world. But at what price? Without attachment to the community and to a higher order of truth, life seems meaningless. The legitimation crisis of modernity indicates that in the long run this victory is self-defeating. The valuation of autonomy does not only threaten the value of authenticity, as has been pointed from the beginning of the Enlightenment by the romantic under- and countercurrent of modernity; it also undermines autonomy itself.

In his theory of social acceleration, Rosa has fleshed out how the modern imperative to control nature has spawned a society that is out of control. Spurred on by an escalatory logic of “dynamic stabilisation”, the synchronisation of all spheres of life by temporal structures has led to acceleration, not only in society (acceleration of economic and technological change), but also of society (acceleration of social change). With the transition from modernity to post-modernity, the hyperacceleration in-and-of society has produced multiple “crises of desynchronisation” that are accompanied by a series of social pathologies: the ecological crisis (the “great acceleration” of the Anthropocene), the democratic crisis (the “great recession” of populism) and the psychocrisis (the “great depression” that followed the Covid-19 crisis) signal that change is too fast for nature, society and subjectivity. Taken together, they are alarming symptoms of a generalised exhaustion of the project of modernity.

The articulation of Taylor’s (2018) theistic-romantic-expressive intuitions about resonance into a mature theory of sympathetic vibrations has reduced the tensions between the three intellectual, moral and spiritual currents of modernity (theism, naturalism and expressivism) to a dual opposition between two modes of world-relation: *Vernehmen* and *Herstellen* (Heidegger), *eros* and *thanatos* (Freud), labour and interaction (Habermas), reification and recognition (Honneth) or alienation and resonance (Rosa). By opening up the *homo clausus* of modernity to its environment, resonance “unbuffers” and “depunctualises” the self, plunging it once again into the stream of life from it which emerges as the conscious tip of evolution that knowingly and willingly collects and connects all beings of the universe into the soul, into the person (from *per-sonare*). The reduction of a tripartite division into a binary smuggles theism in through the back door. The vertical axis of resonance is an axis of transcendence. It does not necessarily posit the existence of a personal God, but it presupposes nevertheless that there’s “something out there” that calls upon the person, resonates within its soul and to which the person responds with gratitude.

Rosa´s work alternates between structural pessimism (the Frankfurt School), cultural conservatism (hermeneutics) and personal optimism (New Age). One senses that the author waivers between the “deep structures of classicism and romanticism” (Gouldner 1975: 323–366) that traverse the social sciences since their emergence in the eighteenth century. Both strive for expression in his work at the same time, balancing each other out, but without ever finding complete unity. Like his forebears in Germany, the Jena brand of systematic romanticism is “searching for a way to be modern without having to reject religion” (id., 325) and “pursuing ‘development’ without endorsing ‘progress’” (id., 326). In his worldview, the romantic principle of self-realisation should have the upper hand over the Enlightenment project of self-determination. In the spirit of Louis Dumont’s (1983: 254–299) logic of “hierarchical complementarity”, the principles of equality and freedom should be hierarchically subordinated to the principles of authenticity and difference to complement the dominance of the former, so that the tensions between cultural holism and normative individualism can be tempered in an unstable equilibrium.

At the turn of the twenty-first century, the reactivation of romantic motifs of the nineteenth century and their continuation in the modernisms of the twentieth century could thus be read and interpreted as a counterhegemonic move within critical theory. From the vantage point of a critical theory that deploys mimetic powers against the domination of instrumental reason, romanticism, aestheticism and atheistic mysticism appear as counter currents not against, but within the fold of modernity. The fusion of Enlightenment and Romanticism in a communitarian critical theory of society might thus indicate a progressive and emancipatory strand within the romantic critique of modernity that renews the indictment of alienation and reification without equating the latter with modernity.

Rosa waivers, however, between accepting and dismissing the “incomplete project of modernity”. He accepts it in his earlier writings, but progressively distances himself from liberal theories on the Left that uphold the value of autonomy. In his more recent work, he has proposed to reopen the discussion on what can be called “Romanticism’s ‘incomplete project’” (Rosa, Henning, Bueno 2021: 1). Like the first generation of the Frankfurt School, he tends to identify autonomy with self-mastery and the control of nature and society, reducing it thereby to instrumental rationality. Alleging that the systemic logics of technological acceleration and capitalist competition undermine the freedom that they were supposed to vouchsafe in the first place, he considers modernity a failure. From the point of view of a critical theory that has not (yet) given up on the promises of the Enlightenment and that criticises reification in the name of Reason, the re-activation of some of the tropes of the ethics of romanticism, the aesthetics of modernism and the politics of post-modernism appears as a form of a “romantic anti-capitalism” and an bohemian critique of alienation (Löwy and Sayre 1992).

## Data Availability

Not applicable.

## Footnotes

1 To avoid the overload of references to Rosa (that risks transforming a family name into a Latin declension: *rosa rosa rosam rosae rosae rosa*… as in Jacques Brel’s eponymous song), all references in Harvard-style that only contain indication of year and publication and page number are to Rosa’s publications. All translations from German or French are by the author.

2 A first version of the article was mainly reconstructive with a minor critique at the end. The two anonymous reviewers of the journal insisted, however, that I work out my critique. I have followed their advice but I remain solely responsible for the end result. I would like to thank the members of the Sociofilo Lab in Rio de Janeiro and the participants of the  Social Theory** Colloquium** at the Max Weber Kolleg in Erfurt for their attentive reading, their comments and their suggestions.
